# Suicide Gene Reveals the Myocardial Neovascularization Role of Mesenchymal Stem Cells Overexpressing CXCR4 (MSC^CXCR4^)

**DOI:** 10.1371/journal.pone.0046158

**Published:** 2012-09-28

**Authors:** Jialiang Liang, Wei Huang, Xiyong Yu, Atif Ashraf, Kishore K. Wary, Meifeng Xu, Ronald W. Millard, Muhammad Ashraf, Yigang Wang

**Affiliations:** 1 Departments of Pathology and Laboratory Medicine and Pharmacology and Cell Biophysics, College of Medicine, University of Cincinnati Medical Center, Cincinnati, Ohio, United States of America; 2 Medical Research Center, Guangdong General Hospital, Guangdong Academy of Medical Sciences, Southern Medical University, Guangzhou, China; 3 Department of Pharmacology, University of Illinois, Chicago, Illinois, United States of America; University of Cincinnati, United States of America

## Abstract

**Background:**

Our previous studies indicated that MSC^CXCR4^ improved cardiac function after myocardial infarction (MI). This study was aimed to investigate the specific role of MSC^CXCR4^ in neovascularization of infarcted myocardium using a suicide gene approach.

**Methods:**

MSCs were transduced with either lentivirus-null vector/GFP (MSC^Null^ as control) or vector encoding for overexpressing CXCR4/GFP. The MSC derived-endothelial cell (EC) differentiation was assessed by a tube formation assay, Dil-ac-LDL uptake, EC marker expression, and VE-cadherin promoter activity assay. Gene expression was analyzed by quantitative RT-PCR or Western blot. The suicide gene approach was under the control of VE-cadherin promoter. *In vivo* studies: Cell patches containing MSC^Null^ or MSC^CXCR4^ were transduced with suicide gene and implanted into the myocardium of MI rat. Rats received either ganciclovir (GCV) or vehicle after cell implantation. After one month, the cardiac functional changes and neovascularization were assessed by echocardiography, histological analysis, and micro-CT imaging.

**Results:**

The expression of VEGF-A and HIF-1α was significantly higher in MSC^CXCR4^ as compared to MSC^Null^ under hypoxia. Additionally, MSC^CXCR4^ enhanced new vessel formation and EC differentiation, as well as STAT3 phosphorylation under hypoxia. STAT3 participated in the transcription of VE-cadherin in MSC^CXCR4^ under hypoxia, which was inhibited by WP1066 (a STAT3 inhibitor). In addition, GCV specifically induced death of ECs with suicide gene activation. *In vivo* studies: MSC^CXCR4^ implantation promoted cardiac functional restoration, reduced infarct size, improved cardiac remodeling, and enhanced neovascularization in ischemic heart tissue. New vessels derived from MSC^CXCR4^ were observed at the injured heart margins and communicated with native coronary arteries. However, the derived vessel networks were reduced by GCV, reversing improvement of cardiac function.

**Conclusion:**

The transplanted MSC^CXCR4^ enhanced neovascularization after MI by boosting release of angiogenic factors and increasing the potential of endothelial differentiation.

## Introduction

Myocardial infarction (MI) occurs when coronary blood supply is interrupted, destroying distal blood vessels and myocardium. Insufficient cardiac capillary density and perfusion after MI have been identified as critical conditions triggering endothelial apoptosis, leading to an increase in infarct size and left ventricular dysfunction. Thus, therapeutic angiogenesis has been proposed as an important strategy for the treatment of vascular insufficiency in MI [1], [2]. Recently, progenitor/stem cell therapy has shown the potential to reverse ischemic damage and repair heart tissue injury through angiogenesis [3], [4]. The multipotency, low immunogenicity, ready availability, and extensive capacity for expansion of bone morrow derived mesenchymal stem/stromal cells (MSCs) has led to their adoption as an important cell resource for regenerative medicine [5], [6]. For decades, transplanted MSCs have been shown to improve angiogenesis after MI, but the mechanism by which this process occurs remains controversial. Emerging evidence demonstrates that the therapeutic effects may result from the growth factors secreted by MSCs, as well as the differentiation into endothelial cells (ECs), pericytes, smooth muscle, and cardiomyocytes (CM) [6]–[8]. Therefore, it is clinically significant to develop approaches that increase the paracrine effects or cardiovascular cell differentiation of MSCs for post-MI therapy. Considering the triple lineage differentiation potential of MSCs, the vascular cell fate decision is particularly important to the restoration of cardiac function after MI [9].

It was initially thought that MSCs differentiate into ECs, which become integrated into the newly formed blood vessels [10]–[12]. However, the vascular differentiation potential of MSCs remains controversial; some studies have suggested that ECs derived from ordinary MSCs are rare and infrequently detected after transplantation [13]–[15]. Alternatively, it has been speculated that angiogenic growth factors released by MSCs (promoting the growth of pre-existing vessels) are directly responsible for the beneficial effects [13], [14]. According to such studies, it is very difficult for ordinary MSCs to differentiate into ECs. However, through genetic engineering, it is possible to enhance both the paracrine effects and the endothelial differentiation potency of MSCs.

In our previous studies, MSCs were genetically engineered to overexpress CXCR4 using viral transduction (MSC^CXCR4^). The mobilization and engraftment capacity of MSC^CXCR4^ into the ischemic area were enhanced, as was the secretion of paracrine factors [e.g., vascular endothelial growth factor-A (VEGF-A)], which promoted neomyoangiogenesis and alleviated early signs of left ventricular remodeling [16]–[18]. However, the mechanisms by which MSC^CXCR4^ promote cytokine secretion and support neovascularization effects remain to be elucidated. In the present study we investigated the pathways relevant to self-renewal or differentiation of MSCs, including hypoxia-inducible factor-1α (HIF-1α) [19], phosphoinositide 3-kinase (PI3K) [20], mitogen-activated protein kinase (MAPK) [21], and the signal transducers and activators of transcription 3 (STAT3) pathway [22].

In addition, the extent to which CXCR4 overexpression alters the tendency of transplanted MSCs to differentiate into ECs has not yet been reported from *in vivo* studies. To assess EC differentiation from MSC^CXCR4^ and the resulting changes in cardiac function after transplantation, an “inducible suicide gene” approach was employed. The herpes simplex viral genome encodes an enzyme, thymidine kinase (TK), which is foreign to mammalian cells [23]. If the TK gene, following a tissue specific promoter (e.g. vascular endothelium (VE)-cadherin), is transduced into the target cells, the TK expression will be under the control of the cell phenotype. TK converts the pro-drug ganciclovir (GCV) into a cytotoxic agent which causes “cell suicide” but has no effect on cells without TK expression [9], [24]. This approach was used to selectively ablate ECs differentiated from MSCs, allowing for direct assessment of the contribution of MSC-to-EC differentiation to cardiac repair, and the degree to which CXCR4 overexpression enhances this process. Thus, by specifically targeting differentiated ECs, we address the role of MSC^CXCR4^ in neovascularization during cardiac repair after MI.

## Materials and Methods

Experiments using animal subjects or animal-derived materials were conducted in accordance with the Guide for the Care and Use of Laboratory Animals (NIH Publication No. 85-23, revised 1996) and under guidelines and protocols approved by the University of Cincinnati Institutional Animal Care and Use Committee.

### Reagents

Cell culture supplies were obtained from Invitrogen (Carlsbad, CA). Antibody against CXCR4 was purchased from Millipore (Temecula, CA). Antibodies against VEGF-A, HIF-1α, GFP, Troponin I, and β-actin were from Santa Cruz Biotechnology (Santa Cruz, CA). LY294002 and antibodies against phosphorylated (p-) STAT3 (Tyr705) and STAT3 were from Cell Signaling Technology (Beverly, MA). WP1066 was purchased from Chemicon (Millipore). Ganciclovir (GCV), SB203580, SP600125, PD98059, and other readily available chemicals were purchased from Sigma-Aldrich (St Louis, MO).

### Lentiviral vectors and transduction

Lentiviral vector backbone pCDH-CMV-MCS-EF1-CopGFP+Puro (pCDH-GFP) was purchased from System Biosciences (Mountain View, CA). The rat CXCR4 CDS was subcloned into the EcoRI and NotI restriction enzyme sites of pCDH-GFP from the reported vector [18] for CXCR4 overexpression (pCDH-CXCR4-GFP). To determine the contribution of MSC^CXCR4^ to angiogenesis, the gene suicide therapy approach was applied. The luciferase gene reporter vector accommodating wild type VE-cadherin promoter (pGL-VE) was kindly provided by Dr. Kishore K. Wary [25]. The multiple cloning site of pCDH-GFP between EcoRI and NotI was replaced with TK gene obtained from pORF-TK (Invivogen, San Diego, CA) to construct the vector pCDH-TK-GFP. VE-cadherin promoter was then subcloned into pCDH-TK-GFP between ClaI and XbaI restriction enzyme sites replacing the CMV promoter (pCDH-VE-TK), while a short fragment (21 bp) was subcloned into the same sites to produce a promoterless vector as the negative control (pCDH-N-TK). (See Table S1 for the sequences of PCR primers for subcloning.) The identity of the promoter and gene was confirmed by sequencing (Genewiz, South Plainfield, NJ). According to manufacturer's instructions (System Biosciences), pseudoviral particles were produced in 293TN cells using pPACK Lentivector Packaging Kit and concentrated by PEG-it Virus Concentration Solution. The target cells were transduced with pseudoviral stock and selected by puromycin (5 µg/mL, Sigma), as reported previously [26].

### Cell culture and suicide gene approach

MSCs from healthy 8-week-old Sprague-Dawley (SD) rats were isolated from bone marrow aspirates using plastic adherence to remove the non-adherent hematopoietic progenitors and blood cells, as described previously [18]. Confluent MSCs in seeded cultures were removed from the flask by 0.25% trypsin. Passage 2–4 MSCs were used in the study. Human umbilical vein endothelial cells (ECs) were purchased from American Type Culture Collection (ATCC, Bethesda, MD) and cultured in endothelial growth medium with 10% fetal bovine serum (FBS). Mouse muscle myoblasts were purchased from ATCC. Cardiomyocytes from hearts of neonatal rats were isolated by collagenase digestion as described previously [27]. Cells were cultured in low glucose Dulbecco's modified Eagle's medium (DMEM) containing 10% FBS and 1% antibiotics (streptomycin and penicillin), incubated at 37°C in a humid atmosphere of 5%CO_2_/95% air (as normoxic control). MSCs were exposed to hypoxic conditions (O_2_/CO_2_ incubator-MCO-18M, Sanyo) in a humidified atmosphere of 1%O_2_/5%CO_2_/94%N_2_. MSC^CXCR4^ were genetically engineered by pCDH-CXCR4-GFP lentiviral transduction, and MSC^Null^ were manipulated by pCDH-GFP lentiviral transduction. When the cell population reached 70–80% confluence, all cell experiments were performed without serum or antibiotics, and repeated at least three times.

Viral supernatants containing pCDH-N-TK or pCDH-VE-TK were incubated to ECs (EC^N-TK^ or EC^VE-TK^) and MSCs (MSC^N-TK^ or MSC^VE-TK^), respectively. And then the cells (MSCs, ECs, cardiomyocytes, and myoblasts) were treated with 100 µM GCV (PBS as vehicle) for 4 days. The number of viable cells identified by GFP or immunostaining was counted under the microscope at 200-magnification.

### Western blotting

MSC^Null^ or MSC^CXCR4^ were exposed to either normoxic or hypoxic conditions. The CXCR4, VEGF-A, HIF-1α, and STAT3 were determined by western blotting, according to the protocols described previously [18]. Briefly, after electrophoresis, transformation, and immunoblotting, the samples on PVDF membranes were visualized using an enhanced chemiluminescence system (Invitrogen), exposed to X-ray film and then quantified by laser scanning densitometry (GE Healthcare Life Sciences, Piscataway, NJ). β-actin was used as the internal reference control of CXCR4, VEGF-A, and HIF-1α expression. STAT3 phosphorylation was analyzed after normalization against total STAT3.

### Quantitative RT-PCR (qPCR)

The mRNA levels of VEGF-A, angiopioetin-1, IGF-1α, VE-cadherin, and CD31 in MSC^Null^ or MSC^CXCR4^ were examined by qPCR according to the protocols reported previously [17]. (The sequences of PCR primers are listed in Table S1.) Briefly, after reverse transcription, PCR was carried out on the iQ5 real-time system (Bio-Rad, Hercules, CA) with the SYBR Supermix (Qiagen, Valencia, CA). The fold changes of each target mRNA expression relative to β-actin under experimental and control conditions were calculated based on the threshold cycle (C_T_) as r = 2^−Δ(ΔC^_T_^)^, where ΔC_T_ = C_T_(target)−C_T_(β-actin) and Δ(ΔC_T_) = ΔC_T_(experimental)−ΔC_T_(control).

### Tube formation assay

The assay was performed with a tube formation assay kit (Chemicon, Millipore) according to the manufacturer's instructions. Briefly, the solution of ECMatrix was thawed on ice overnight, mixed with 10 diluents of ECMatrix, and placed in a 96-well tissue culture plate at 37°C for 1 hour to allow the matrix solution to solidify. MSC^Null^ or MSC^CXCR4^ were exposed to either normoxia or hypoxic conditions for 48 hours. They were then seeded (5×10^3^cells/well) on top of the solidified matrigel and incubated at 37°C for 4 hours. After cellular network structures were fully developed, the total capillary tube number was measured under an inverted light microscope (Olympus America, Melville, NY) at 200-magnification. Four independent fields were assessed for each well, and the average number of tubes was determined.

### Identification of the MSC-derived ECs

MSC^Null^ and MSC^CXCR4^ were exposed to either normoxia or hypoxic conditions for 48 hours. Adherent MSCs were incubated with 200 µg/ml 1,1-dioctadecyl-3,3,3,3-tetramethyl-indocarbocyanine-labeled acetylated low density lipoprotein (Dil-Ac-LDL, Biomedical Technologies) for 4 hours and then fixed with 4% paraformaldehyde for 10 min. The positive cells stained with Dil-Ac-LDL were identified as differentiated ECs with a fluorescent microscope (Olympus) at 200-magnification. Nuclei were stained with DAPI. The number of positive cells per well was counted in 4 randomly selected fields.

### Transient transfection and luciferase assay

To determine the effect of CXCR4 overexpression on VE-cadherin transcription, MSC^Null^ and MSC^CXCR4^ were transiently transfected with pGL-VE and pGL4.75 (*Renilla* luciferase control vector, Promega, Madison, WI) by DharmaFECT® Duo transfection reagent (Thermo Scientific, Florence, KY) according to the manufacturer's protocol, and then exposed to either normoxia or hypoxic conditions for 24 hours respectively. Additionally, to explore the pathways related to the VE-cadherin promoter activity, MSC^CXCR4^ were transiently transfected with pGL-VE and pGL4.75 and then treated with DMSO (vehicle), SB203580 (p38 inhibitor, 5 µM), SP600125 (JNK inhibitor, 5 µM), PD98059 (ERK inhibitor, 10 µM), LY294002 (PI3K inhibitor, 10 µM), or WP1066 (STAT3 inhibitor, 5 µM) for 1 hour respectively, before exposure to hypoxia for 24 hours. Subsequently, we used a Dual-Glo™ luciferase assay system (Promega) to detect the promoter activity according to the manufacturer's protocol. After incubation, cells were lysed and double luciferase activity was measured using a luminometer (Mutilabel detection platform, Bioscan, Inc. Washington, DC). *Firefly* luciferase activity was normalized by *Renilla* luciferase activity in cell lysates, and at least three independent experiments were repeated.

### Cell transplantation and MI model

The stable cell line MSC^VE-TK^ was transduced using control or CXCR4 lentivirus to generate MSC^TK+Null^ or MSC^TK+CXCR4^ respectively. MI model after permanent LAD ligation was developed in SD rats as described previously [17], [18]. Meanwhile, the MI rats were implanted with a peritoneal cell patch as described previously [16], [17], containing MSC^TK+Null^ or MSC^TK+CXCR4^ (1×10^6^). After one week, the rats were injected intraperitoneally with 10 mg/kg GCV (Sigma) daily for 7 days and the same dose of saline was injected as vehicle controls. Briefly, the different experimental groups were divided as follows: (i) MSC^TK+Null^ with vehicle, (ii) MSC^TK+CXCR4^ with vehicle, (iii) MSC^TK+Null^ with GCV, (iv) MSC^TK+CXCR4^ with GCV. Since MSCs transduced with lentiviral pCDH-N-TK or pCDH-VE-TK had the similar effects on the cardiac neovascularization and functional restoration of MI rats, the data of MSCs transduced with lentiviral pCDH-N-TK are not included in the present study.

### Cardiac function assessment by echocardiography

Echocardiography (iE33 Ultrasound System, Phillips, Oceanside, CA) with a 15-MHz probe was performed at 4 weeks after MI to assess systolic and diastolic dimensions in addition to anterior wall thickness. Hearts were imaged in 2-D long-axis view at the level of the greatest left ventricular (LV) diameter with animals under light general anesthesia. This view was used to position the M-mode cursor perpendicular to the LV anterior and posterior walls. LV end-diastolic (LVDd) and end-systolic diameters (LVDs) were measured from M-mode recordings using the leading-edge method. LV ejection fraction (EF) was calculated as: EF = (LVDd^3^−LVDs^3^)/LVDd^3^×100%. Fractional shortening (FS) was also determined as: FS = (LVDd−LVDs)/LVDd×100%. All measurements were performed according to the American Society for Echocardiography leading-edge technique, and averaged over three consecutive cardiac cycles.

### Immunohistochemical analysis

The neovascularization derived from MSCs was identified by von Willebrand Factor (vWF) and GFP expression. The immunohistochemical studies were performed on heart tissues at 4 weeks after MI. Briefly, heart tissue sections were harvested, fixed in 10% formalin, and sectioned at 5 µm thickness. The antibodies against vWF or GFP were used to discern new vessel networks derived from MSCs. DAPI was used to identify nuclei. Fluorescent imaging was performed with the microscope at 200-magnification. The vWF or GFP positive vessel numbers per mm^2^ were counted in 4 randomly selected fields.

### Measurement of infarct size

Fixed hearts were embedded in paraffin and sections from apex, mid-LV, and base were stained with Masson's Trichrome. BX41 camera was used to obtain images of LV area on each slide using MagnaFire software (Olympus). Infarct size and total LV area of each image were measured using the Image-Pro Plus software (Media Cybernetics, Carlsbad, CA), and the percentage of the infarct size was calculated using the formula [fibrosis area/total LV area]×100%, as previously described [17], [27].

### Micro Computed Tomography (micro-CT) imaging

At 4 weeks after MI, the animals were deeply anesthetized (100 mg/kg ketamine and 10 mg/kg xylazine) and anticoagulated with heparin saline (100 IU/kg). The thoracic cage was opened and the thoracic aorta isolated, into which a polyethylene cannula was inserted. The arterial cannula was connected to a syringe pump, and heparin was infused at constant pressure (100 mmHg) and flow (200 µL/min). Before perfusion, the superior vena cava and inferior vena cava (IVC) were opened as vents. All animals were euthanized with i.v. KCl solution (25 mmol/L), to stop the heart in diastole. Then, MICROFIL silicone rubber, including 8 mL MV-compound (MICROFIL MV-122, Flow Tech, South Windsor, CT), 10 mL MV-diluents, and 5% MV curing agent, was infused into the coronary arteries via the aorta. The MICROFIL perfusion was continued until the materials flowed freely from the IVC. The heart then underwent a Micro-CT imaging scan on Inveon Multimodality System (Siemens, Malvern, PA). We performed image segmentation to define the patch area and its blood vessels, followed by quantitative analysis of blood vessel volume using the Inveon workstation and software.

### Statistical Analysis

Data from repeated experiments are present as the mean ± SD. Data were compared between groups using one- or two-way analysis of variance (ANOVA). Following ANOVA, the least significant difference (LSD) post hoc test or Dunnetts t-test with Bonferroni correction were used to analyze the significance of differences between mean values of the experimental and control groups. *p*<0.05 was considered significant.

## Results

### MSC^CXCR4^ enhanced the expression of VEGF-A and HIF-1α under hypoxia

**Figure 1:**
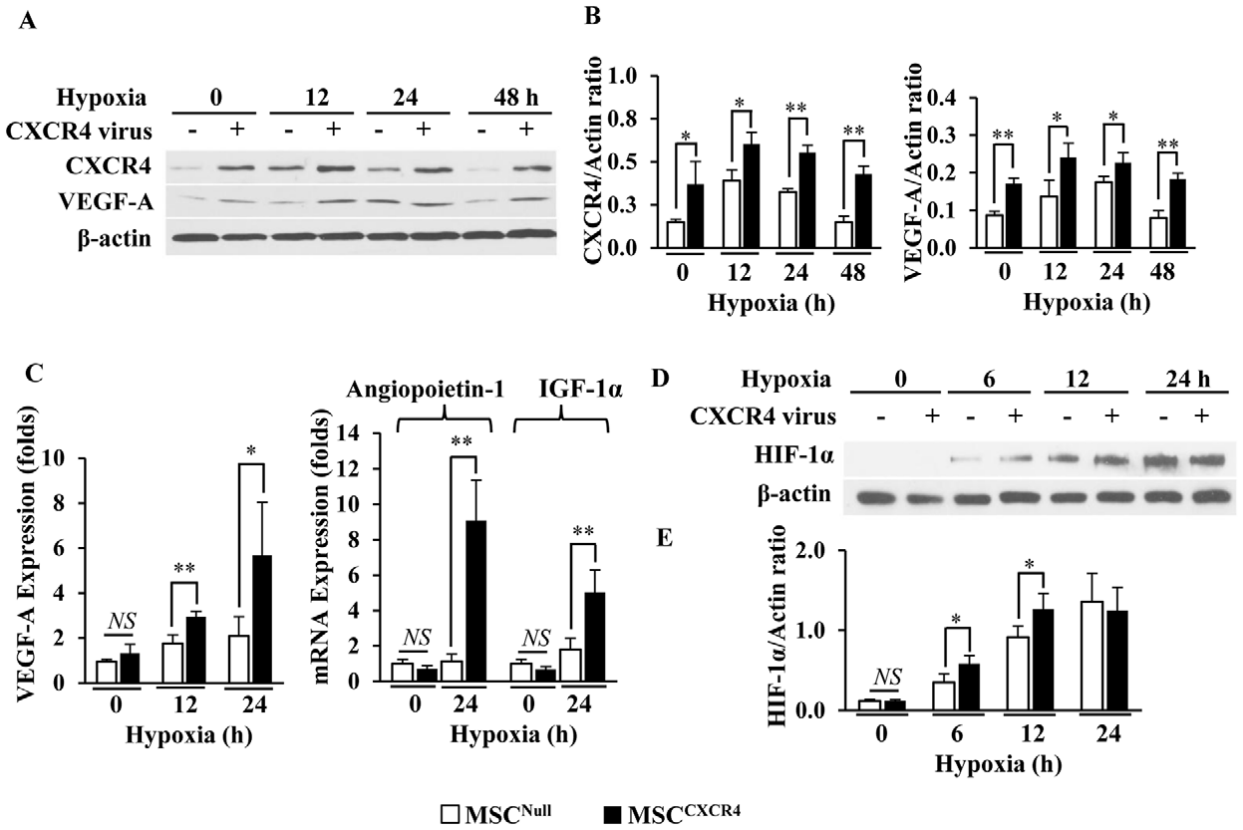
The expression of VEGF-A and HIF-1α under normoxia or hypoxia. (A): The expression of CXCR4 and VEGF-A was analyzed by Western blots in MSCs under normoxia or hypoxia for 48 hours. (B): Quantitative analysis for CXCR4 and VEGF-A expression after normalization against β-actin. (C): The mRNA expression of VEGF-A, angiopoietin-1, and IGF-1α was analyzed by qPCR in MSCs under normoxia or hypoxia for 12 or 24 hours after normalization. (D): The expression of HIF-1α was analyzed by Western blot in MSCs under normoxia or hypoxia for 24 hours. (E): Quantitative analysis for HIF-1α expression after normalization against β-actin. Hypoxia (0 hr), normoxia; CXCR4 virus (−), MSC^Null^; CXCR4 virus (+), MSC^CXCR4^. * *p*<0.05; ** *p*<0.01. Data are the mean ± SD (n = 4).

The level of CXCR4 expression was significantly higher under normoxia in MSC^CXCR4^ when compared to MSC^Null^ (*p*<0.05), and further increased after exposure to hypoxia for 12 to 48 hrs, which was concomitant with an increase in VEGF-A expression confirmed by Western blot (Fig. 1A and 1B). In addition, there was no significant difference in VEGF-A expression between MSC^CXCR4^ and MSC^Null^ under normoxia (Fig. 1C). However, the VEGF-A expression was significantly upregulated in MSC^CXCR4^ as compared to MSC^Null^ (2 fold, *p*<0.05) under hypoxia within 24 hrs, and a similar pattern was also observed in angiopoietin-1 and IGF-1α as analyzed by qPCR (Fig. 1C).

The expression of HIF-1α was scarcely observed in both MSC^Null^ and MSC^CXCR4^ under the normoxic conditions (Fig. 1D). However, the level of HIF-1α was gradually and significantly upregulated from 6 to 24 hrs after exposure to hypoxia in MSC^CXCR4^ as compared to MSC^Null^ (Fig. 1D and 1E).

### Overexpression of CXCR4 enhanced the angiogenesis in MSCs

**Figure 2:**
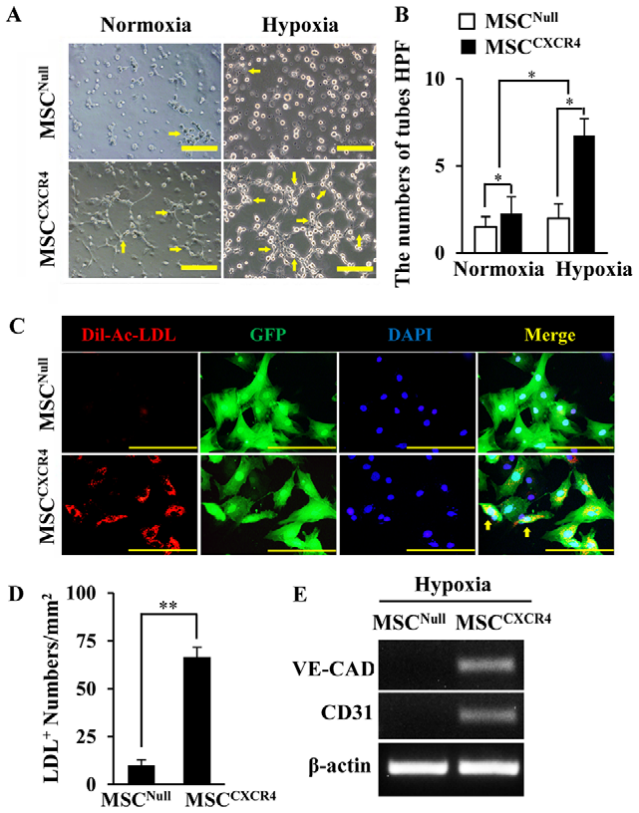
The *in vitro* angiogenic properties of MSC^Null^ and MSC^CXCR4^. (A): The new tube formation of MSCs under normoxia or hypoxia is showed (yellow arrows). Scale bars = 200 µm. (B): Quantification of total tube numbers in high power fields (HPF) was calculated. (C): The EC differentiation under hypoxia is identified by the uptake of Dil-ac-LDL (red); Viral manipulated MSCs are showed by GFP (green); All nuclei are stained with DAPI (blue). Scale bars = 100 µm. (D): Quantification of positive Dil-ac-LDL cell numbers per mm^2^ was calculated. (E): The expression of endothelial specific markers (VE-cadherin and CD31) was analyzed by RT-PCR in MSC^CXCR4^ under hypoxia for 72 hours. CAD, cadherin. * *p*<0.05; ** *p*<0.01. Data are the mean ± SD (n = 4).

The tube formation assay was performed on matrigel-precoated wells. The MSC^Null^ exhibited small round shapes, isolated cells, and minimal migration under both normoxia and hypoxia (Fig. 2A). However, MSC^CXCR4^ led to the development of capillary tubes, sprouting of new capillaries, and finally the formation of cellular networks. The number of tube-like structures was significantly increased in MSC^CXCR4^ as compared to MSC^Null^ under normoxia, and further enhanced by hypoxia (*p*<0.05, Fig. 2A and 2B).

The ECs derived from MSC^CXCR4^ were recognized as cells with endothelial-like spindle-shaped morphology and were identified as Dil-Ac-LDL uptake (Fig. 2C). The number of Dil-Ac-LDL-positive cells was higher in MSC^CXCR4^ as compared to MSC^Null^ (*p*<0.01, Fig. 2D). qPCR revealed that the endothelial markers, VE-cadherin and CD31, were significantly upregulated in MSC^CXCR4^ as compared to MSC^Null^ under hypoxic conditions (Fig. 2E).

### MSC^CXCR4^ enhanced the transcription of VE-cadherin through STAT3 pathway

**Figure 3:**
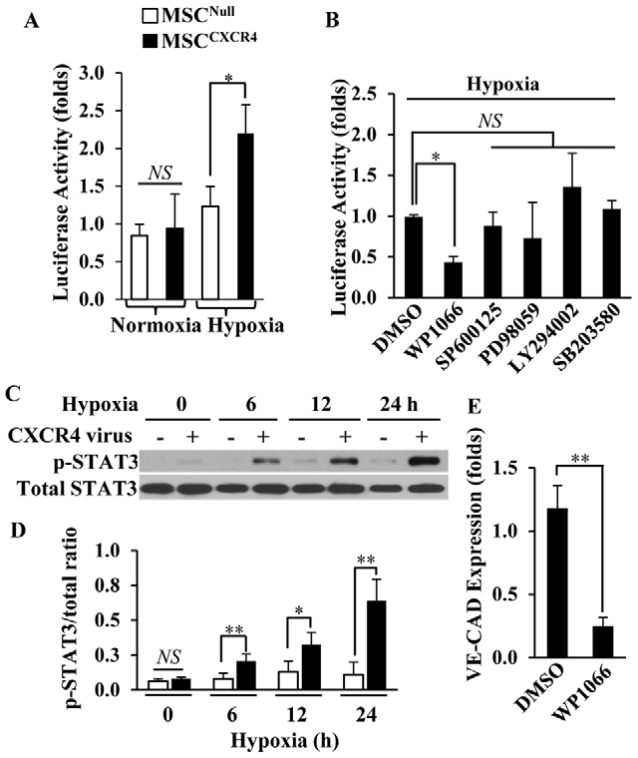
The transcriptional level of VE-cadherin in MSC^CXCR4^ under hypoxia is mediated by STAT3 pathway. (A): The luciferase activity of both MSC^Null^ and MSC^CXCR4^ under normoxia or hypoxia for 24 hours was measured. (B): The VE-cadherin promoter activity of MSC^CXCR4^ under hypoxia for 24 hours was impacted by various inhibitors. (C): STAT3 expression was analyzed by Western blots in MSCs under normoxia or hypoxia for 24 hours. (D): Quantitative analysis for STAT3 phosphorylation after normalization against total STAT3. (E): The VE-cadherin expression was analyzed by RT-PCR in MSC^CXCR4^ treated with DMSO or WP1066 under hypoxia for 72 hours. * *p*<0.05; ** *p*<0.01. All values are expressed as mean ± SD (n = 4).

Under normoxic conditions, the luciferase activity of both MSC^Null^ and MSC^CXCR4^ was low, but it was significantly higher (over 2.5-fold) in MSC^CXCR4^ compared to MSC^Null^ under hypoxia (*p*<0.05, Fig. 3A). Compared to the DMSO treatment, the STAT3 inhibitor (WP1066) significantly decreased the luciferase activity of MSC^CXCR4^ by 60% (*p*<0.05), but other signaling pathway inhibitors had no significant effects (Fig. 3B). After 6 hours of hypoxia, the level of phosphorylated STAT3 gradually increased significantly in MSC^CXCR4^ as compared to the MSC^Null^ (Fig. 3C and 3D). Moreover, WP-1066 treatment significantly reduced the VE-cadherin mRNA expression of MSC^CXCR4^ by 70% (*p*<0.01), as compared to MSC^CXCR4^ treated with DMSO (Fig. 3E).

### Transduction of TK for suicide gene delivery

**Figure 4:**
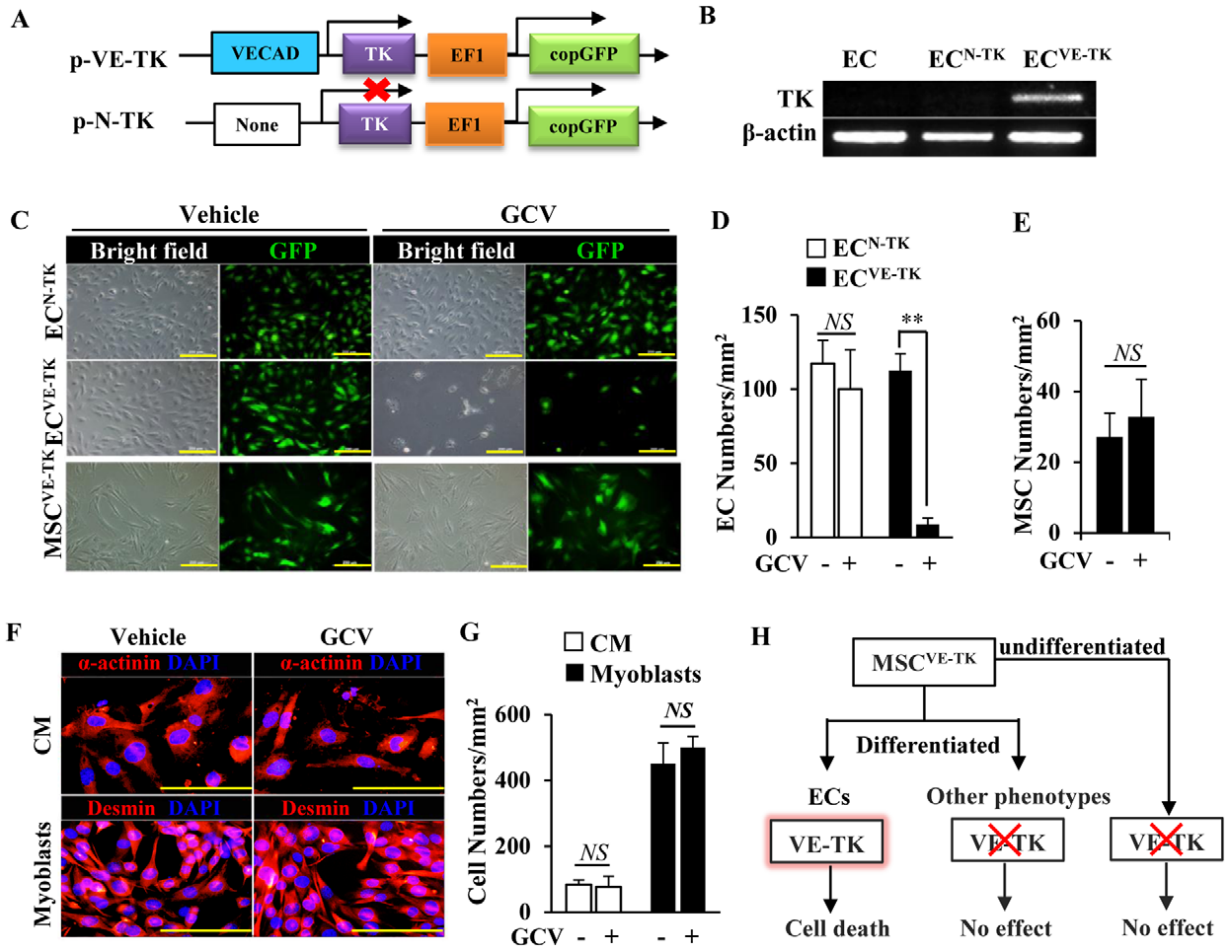
Transduction of TK for suicide gene delivery and the effect of GCV on cell growth. (A): Schematic diagram of suicide gene vector p-VE-TK and its promoterless control vector p-N-TK; VECAD, VE-cadherin promoter; TK, HSV1-thymidine kinase; EF1, constitutive elongation factor 1 α promoter; copGFP, copepod green fluorescent protein. (B): The TK gene was expressed in EC^VE-TK^, but not in ordinary ECs or EC^N-TK^, as analyzed by RT-PCR. (C): Viral manipulated ECs are showed by GFP (green) and contrast ECs in the bright field of light microscopy. Scale bars = 200 µm. (D): Quantification of viable EC numbers. (E): Quantification of viable MSC numbers. (F): Effect of GCV on cardiomyocytes identified by α-actinin (red) and myoblasts identified by desmin (red). All nuclei are stained with DAPI (blue). Scale bars = 100 µm. (G): Quantification of viable cardiomyocyte and myoblast numbers. (H): Schematic diagram for showing that GCV can induce the cell death of ECs derived from MSCs with TK expression which is inactivated in other phenotype or undifferentiated cells. CM, cardiomyocytes; GCV (−), vehicle; GCV (+), ganciclovir 100 µM. ** *p*<0.01. All values are expressed as mean ± SD (n = 4).

The lentiviral vectors pCDH-N-TK and pCDH-VE-TK were constructed, as shown in Figure 4A. The VE-cadherin promoter activity of pCDH-VE-TK (blue) was detected in ECs as the positive control, and the position was removed to produce a promoterless vector pCDH-N-TK as the control vector. The performance of vector was confirmed by GFP expression (Fig. 4C). TK gene can be expressed in EC^VE-TK^, but not in ordinary EC or EC^N-TK^ (Fig. 4B).

The number of EC^VE-TK^ was significantly decreased by the administration of GCV (vs. vehicle, *p*<0.01), but EC^N-TK^ were unaffected (Fig. 4C and 4D). In absence of GCV, there was no significant difference between the cell numbers of EC^VE-TK^ and EC^N-TK^, which indicated that the expression of TK had no effect on EC growth (Fig. 4C and 4D). Moreover, the growth of MSC^VE-TK^ without TK expression under normal culture conditions was not influenced (Fig. 4C and 4E). In addition to MSCs, GCV had no effect on the other non-endothelial cells, including cardiomyocytes and myoblasts (Fig. 4F and 4G). Thus, in the presence of GCV, cell suicide was induced under the control of VE-cadherin promoter, which was not activated in other phenotype or undifferentiated cells (Fig. 4H).

### GCV reversed MSC^TK+CXCR4^ induced neovascularization

**Figure 5:**
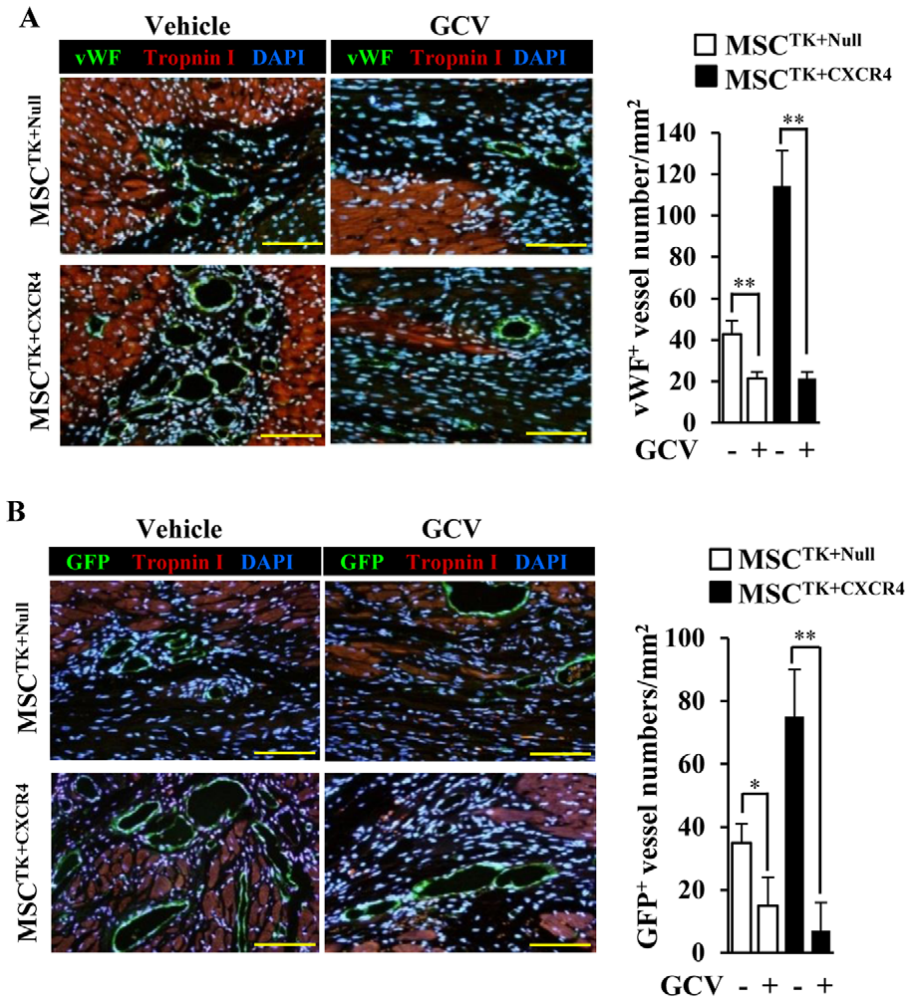
GCV reverses the MSC-induced vessel formation in border area of ischemic heart. (A): The vessel formation in the ischemic border zone is identified by vWF; Cardiomyocytes are identified by troponin I; All nuclei are stained with DAPI; Quantification of vWF positive vessel numbers was analyzed. (B): The new vessels derived from exogenous genetically manipulated MSCs are identified by GFP (green); Cardiomyocytes are identified by troponin I; All nuclei are stained with DAPI. Quantification of GFP positive vessel numbers was calculated. Scale bars = 100 µm. GCV (−), vehicle; GCV (+), ganciclovir 10 mg/kg. * *p*<0.05; ** *p*<0.01. Data are the mean ± SD (n = 5).

In the absence of GCV (Vehicle), MSC^CXCR4^ significantly enhanced the new vessel formation with vWF expression compared to the MSC^TK+Null^ group (Fig. 5A). However, the administration of GCV reduced the vessel numbers of MSC^TK+Null^ (*p*<0.01), especially for the MSC^TK+CXCR4^ (*p*<0.01), as compared to the vehicle treatment (Fig. 5A).

Additionally, the number of GFP positive new vessels derived from MSC^CXCR4^ was significantly higher than that of MSC^TK+Null^ in the absence of GCV (Fig. 5B). In contrast, GCV abolished the new vessel formation derived from MSC^TK+Null^, as observed that GFP^+^ cell number was significantly reduced (*p*<0.05), especially for the MSC^TK+CXCR4^ (*p*<0.01), as compared to the vehicle treatment (Fig. 5B). GCV had no effect on the normal vascular structures in cardiac tissues of rats (data not shown).

### Neovascularization integrating the cell patch and cardiac circulation

**Figure 6:**
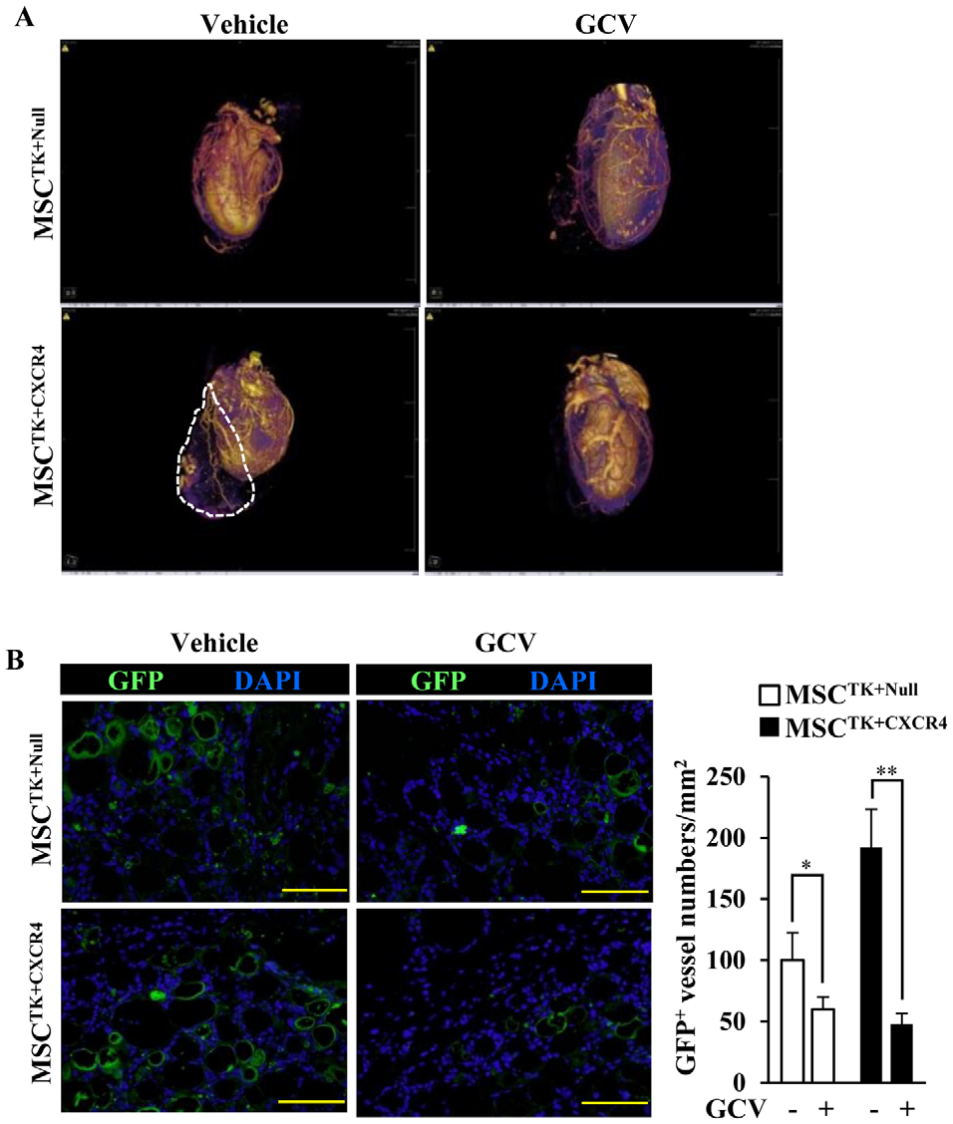
Micro-CT scan shows the vessel distribution in the whole heart. (A): The perfusion of contrast agents indicates the cardiac vessel networks of various groups. The dotted line shows the location of cell patch. (B): In cell patch, the new formed vessels are identified by GFP (green) and quantitative data. All nuclei are stained with DAPI (blue). Scale bars = 100 µm. * *p*<0.05; ** *p*<0.01. All values are expressed as mean ± SD (n = 4).

The neovascularization of implanted MSC patches was further analyzed by micro-CT scan (Video S1). The radiocontrast agents which were infused into the coronary arteries showed the new vessel networks (Fig. 6A). The vessels derived from MSCs in cell patch were identified by GFP (Fig. 6B). In the absence of GCV, the new vessels from MSC^TK+Null^ group were seldom identified (Fig. 6A and 6B). However, a significantly increased number of new vessels derived from MSC^TK+CXCR4^ were observed (Fig. 6A and 6B), which were integrated with the coronary arteries in the heart (indicated by dotted line, Fig. 6A). Importantly, the new vessels derived from MSC^TK+CXCR4^ were eliminated by GCV treatment (Fig. 6A and 6B). Therefore, the reversal effect of GCV revealed that CXCR4 overexpression increased the potential and tendency of transplanted MSC differentiated into ECs.

### GCV reversed the CXCR4-mediated improvement in cardiac function by destroying ECs derived from transplanted MSCs

**Figure 7:**
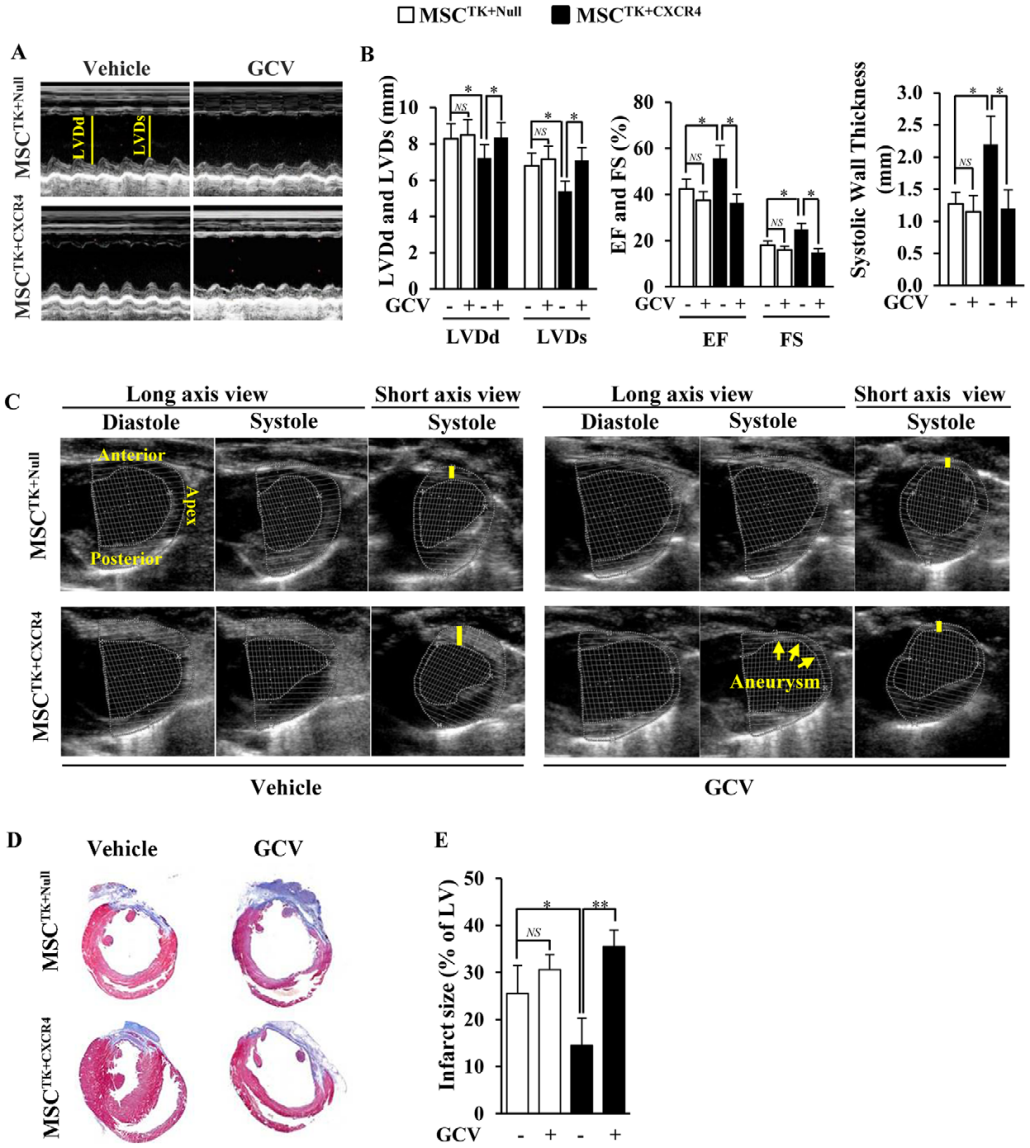
Effect of GCV on cardiac function and heart remodeling. (A): M-mode echocardiograms in various treatment groups at 4 weeks after cell patch implantation. (B): Left ventricular end-diastolic dimension (LVDd), left ventricular end-systolic dimension (LVDs), ejection fraction (EF), fractional shortening (FS), and systolic wall thickness of various groups. (C): The long/short axis views of various groups. Left ventricular wall thinning (yellow bar) and aneurysm formation (yellow arrows). (D): 5-µm sections from heart slices stained with Masson's Trichome in various treatments. (E): The percentage of infarct size in hearts of various groups. * *p*<0.05; ** *p*<0.01. All values are expressed as mean ± SD (n = 5).

Cardiac function was analyzed by echocardiography after cell patch implantation for 4 weeks. Compared to the MSC^TK+Null^ group, the MSC^TK+CXCR4^ group exhibited a significant reduction in LV remodeling, as showed by decreased LVDd and LVDs (*p*<0.05, as well as increased systolic wall thickness of MSC^TK+CXCR4^ group, Fig. 7A to 7C). Additionally, EF and FS of the MSC^TK+CXCR4^ group was significantly higher than that of MSC^TK+Null^ group (*p*<0.05, Fig. 7B).

Functional assessments (LVDd, LVDs, LVEF, LVFS, and systolic wall thickness) were then used to determine the effect of GCV on restoration of left ventricular mechanics. In the MSC^TK+Null^ group, there was no statistical difference in cardiac remodeling between vehicle and GCV treatments (Fig. 7A and 7B). However, the beneficial effects of the MSC^TK+CXCR4^ on heart function were significantly reversed after the administration of GCV, as indicated by a significant increase in both LVDd and LVDs, and an accompanying reduction of EF, FS, as well as systolic wall thickness as compared to the vehicle treatment (Fig. 7A to 7C).

In addition, the MSC^TK+Null^ transplanted animals in both vehicle and GCV treatment groups exhibited left ventricular wall thinning (Fig. 7C). However, in the absence of GCV, left ventricular wall thinning was significantly preserved in the MSC^TK+CXCR4^ group, as compared to MSC^TK+Null^ group (Fig. 7C). Importantly, when the MSC^TK+CXCR4^ group underwent GCV treatment, the left ventricular wall thickness was significantly reduced as compared to vehicle treatment in same group, and aneurysm formation was also observed in some hearts (Fig. 7C).

The change of LV infarct size was assessed using Masson's Trichrome staining (Fig. 7D and 7E). In the absence of GCV, the infarct size was significantly reduced in the MSC^TK+CXCR4^ group as compared to MSC^TK+Null^ group (*p*<0.05). Additionally, the therapeutic effect of MSC^TK+CXCR4^ was eliminated after GCV treatment as compared to the vehicle treatment (*p*<0.01).

## Discussion

In this study, we investigated the role of MSC^CXCR4^ in neovascularization during cardiac repair after MI and its mechanism. The *in vitro* studies showed that MSC^CXCR4^ released angiogenic factors and enhanced the capacity for vessel formation under hypoxic conditions, which involved HIF-1α and STAT3 pathways. For the *in vivo* studies, MSC^CXCR4^ seeded on peritoneum (patch) promoted neovascularization when applied to the epicardial surface of MI rats. However, the specific elimination of ECs derived from MSC^CXCR4^ by suicide gene activation significantly abrogated the enhanced capillary density and the improvement of cardiac function.

The autocrine/paracrine mechanism of stem cell therapy plays an important role in cardiac functional restoration after MI [6], [8], [28]. We found that CXCR4 overexpression enhanced the gene expression of VEGF-A in MSCs under hypoxia, which was consistent with observations from our previous studies [17]. VEGF signaling pathway plays an essential role in vascular homeostasis and in the angiogenic cascade [29]. In addition, hypoxia is an important component of an ischemic insult. It is a critical regulator of both protective and pathological vascular adaptations and composes the niche to maintain stem cells [30], [31]. The oxygen-sensing HIF is also important for vascular homeostasis responding to hypoxic conditions [32]. As other studies have demonstrated, the ubiquitin-mediated proteolysis of HIF is inhibited under hypoxia, thereby activating distinct angiogenic factor genes such as VEGF, whose promoters include hypoxia-response elements [30], [33]. In our studies, hypoxia increased the expression of HIF-1α, which was further enhanced by MSC^CXCR4^. Thus, when MSC^CXCR4^ were implanted in a hypoxic microenvironment, the increasing expression of angiogenic factors initiated a cascade that promotes cytokine-induced cardiac angiogenesis.

In addition to the paracrine effect of MSCs, the endothelial differentiation potential also plays an important role in new vessel formation. MSC^CXCR4^ acquired endothelial characteristics, including tube formation, uptake of Dil-ac-LDL, and expression of the endothelial cell markers, suggesting that CXCR4 overexpression enhanced the EC differentiation of MSCs. Within the cadherin family, VE-cadherin is the only specific endothelial adhesion molecule and the major determinant of EC contact integrity and activity, which is very important for vascular development and differentiation [34], [35]. The CXCR4 overexpression enhanced the expression of VE-cadherin at the transcriptional level in MSCs as well as the phosphorylation of STAT3 under hypoxia. The observation was further confirmed by the STAT3 inhibitor (WP1066) which decreased the promoter activity and mRNA expression of VE-cadherin in MSC^CXCR4^ under hypoxia. Therefore, STAT3 participated in the differentiation of MSC^CXCR4^ into ECs by regulating the endothelial gene expression.

CXCR4 is a G protein-coupled receptor and activates several G-protein mediated downstream signaling pathways after binding its ligands [36]. However, the expression of CXCR4 in MSCs showed a gradually decreasing tendency after the hypoxic stimuli over a long timeframe. The decrease in the expression of CXCR4 might result from the ligand-induced receptor internalization and endocytosis via a clathrin or caveolae dependent pathway [37], [38]. Therefore, the overexpression of CXCR4 is important to compensate for pathological insufficiency and prolong the therapeutic effect under the ischemic conditions. One endpoint of CXCR4 signaling is the activation of transcription factors [36]. In addition, the SDF-1α/CXCR4 axis was reported to upregulate STAT3 activation in ischemic cardiomyocytes, thereby mediating acute cardioprotection [39]. The activation of STAT3 also played a pivotal role in EC differentiation of cardiac stem cells [40]. Consequently, under the hypoxic microenvironment, along with the secretion of angiogenic factors, the overexpression of CXCR4 activated the STAT3 signaling pathway in MSCs, thereby promoting the differentiation into ECs.

To highlight the role of ECs derived from MSCs in myocardial neovascularization, the effect of MSC^CXCR4^ on angiogenesis was abolished by the suicide gene approach under the control of an endothelial specific promoter. The transcription and expression of VE-cadherin in ECs are due to its promoter containing multiple specific regulatory elements, which are silenced in non-endothelial cells [35], [41], [42]. Hence, the transduction of TK gene linked to VE-cadherin promoter can achieve the suicide gene approach. After GCV is absorbed by ECs derived from MSCs, it can be transformed into a cytotoxic agent by the activation of TK gene and cause cell death [23], [24]. Our data showed that TK was expressed under the control of VE-cadherin promoter in ECs. GCV specifically killed ECs expressing TK, indicating the effectiveness of the suicide gene approach. However, GCV had no effect on the growth of non-endothelial cells, including ordinary MSCs, myoblasts, and normal preexisting cardiovascular cells.

By using the GCV-induced suicide gene approach, we assessed the EC differentiation level of MSCs after implantation of cell patches. The CXCR4 overexpression enhanced the migration and vessel formation of MSCs in the infarcted area, which was consistent with our previous studies, in which we showed significantly enhanced heart function [16], [17]. However, the administration of GCV decreased the vessel formation of MSC^CXCR4^, and abolished the benefits of MSC^CXCR4^ induced cardiac protection. As revealed by micro-CT, the vessel networks derived from MSC^CXCR4^ were observed around the left ventricle and in the cell patch, which communicated with the native coronary arteries. The vascular integration provides oxygen and nutrient supplies to the myocardium served by the vessels [43]. We confirmed that the derived new vessels around the ischemic heart and in the cell patch were eliminated by GCV treatment.

To assess the therapeutic effect of ECs derived from MSCs, cardiac function improvement was analyzed using echocardiography combined with the suicide gene approach. In the absence of GCV, MSC^CXCR4^ ameliorated the post-MI loss of cardiac function as indicated by increased left ventricular ejection fraction and decreased the extent of remodeling after MI. However, the administration of GCV reversed the beneficial effects of MSC^CXCR4^, as evidenced by worsening cardiac function, aneurysm formation, and left ventricular wall thinning. Clearly, GCV reversed the heart functional improvement after cell implantation, highlighting that the therapeutic effects of MSC^CXCR4^ were mainly resulted from differentiated ECs to form new vessels. The therapeutic effects were significantly reversed when the suicide gene activation was invoked to specifically kill the MSC^CXCR4^ differentiated into ECs, suggesting the important role of MSC-to-EC differentiation in MI treatment. The EC differentiation of MSCs was enhanced by CXCR4 overexpression, which contributed to the neovascularization in the ischemic hearts. Importantly, ECs are the critical component of blood vessels, regulating angiogenic sprouting and vascular tube formation [44]. Thus, the new vessels derived from MSC^CXCR4^ can improve blood flow for supplying nutrition to the damaged heart, thereby promoting the cardiac functional restoration.

### Conclusions

In summary, MSC^CXCR4^ enhanced the release of angiogenic factors and the EC differentiation of MSCs involved in HIF-1α and STAT3 pathways under hypoxia. The specific gene suicide approach demonstrated that the neovascularization of MSC^CXCR4^ in the ischemic hearts was mainly due to the EC differentiation. The new vessels derived from MSC^CXCR4^ integrated to coronary artery, providing new functional vascularity for ischemic tissues.

## Supporting Information

Table S1 **The sequences of PCR primers.** F, forward primer; R, reverse primer.
(DOCX)

Video S1 **The micro-CT imaging of new vessels derived from MSC^CXCR4^.** The video shows the outline of the whole heart which is surrounded with the new formed vessels from the MSC^CXCR4^ patch.
(WMV)

## References

[1] Rivard A, Isner JM (1998) Angiogenesis and vasculogenesis in treatment of cardiovascular disease. Mol Med 4: 429–440. 9713822 PMC2230333

[2] Ziebart T, Yoon CH, Trepels T, Wietelmann A, Braun T, et al (2008) Sustained persistence of transplanted proangiogenic cells contributes to neovascularization and cardiac function after ischemia. Circ Res 103: 1327–1334. 18927463 10.1161/CIRCRESAHA.108.180463

[3] Ripa RS, Haack-Sorensen M, Wang Y, Jorgensen E, Mortensen S, et al (2007) Bone marrow derived mesenchymal cell mobilization by granulocyte-colony stimulating factor after acute myocardial infarction: results from the Stem Cells in Myocardial Infarction (STEMMI) trial. Circulation 116: I24–I30. 17846310 10.1161/CIRCULATIONAHA.106.678649

[4] Schachinger V, Erbs S, Elsasser A, Haberbosch W, Hambrecht R, et al (2006) Intracoronary bone marrow-derived progenitor cells in acute myocardial infarction. N Engl J Med 355: 1210–1221. 16990384 10.1056/NEJMoa060186

[5] Jiang YH, Jahagirdar BN, Reinhardt RL, Schwartz RE, Keene CD, et al (2002) Pluripotency of mesenchymal stem cells derived from adult marrow. NATURE 418: 41–49. 12077603 10.1038/nature00870

[6] Nombela-Arrieta C, Ritz J, Silberstein LE (2011) The elusive nature and function of mesenchymal stem cells. Nat Rev Mol Cell Biol 12: 126–131. 21253000 10.1038/nrm3049 PMC3346289

[7] Quevedo HC, Hatzistergos KE, Oskouei BN, Feigenbaum GS, Rodriguez JE, et al (2009) Allogeneic mesenchymal stem cells restore cardiac function in chronic ischemic cardiomyopathy via trilineage differentiating capacity. Proc Natl Acad Sci U S A 106: 14022–14027. 19666564 10.1073/pnas.0903201106 PMC2729013

[8] Gnecchi M, Zhang Z, Ni A, Dzau VJ (2008) Paracrine mechanisms in adult stem cell signaling and therapy. Circ Res 103: 1204–1219. 19028920 10.1161/CIRCRESAHA.108.176826 PMC2667788

[9] Yoon CH, Koyanagi M, Iekushi K, Seeger F, Urbich C, et al (2010) Mechanism of improved cardiac function after bone marrow mononuclear cell therapy: role of cardiovascular lineage commitment. Circulation 121: 2001–2011. 20421519 10.1161/CIRCULATIONAHA.109.909291

[10] Oswald J, Boxberger S, Jorgensen B, Feldmann S, Ehninger G, et al (2004) Mesenchymal stem cells can be differentiated into endothelial cells in vitro. Stem Cells 22: 377–384. 15153614 10.1634/stemcells.22-3-377

[11] Kocher AA, Schuster MD, Szabolcs MJ, Takuma S, Burkhoff D, et al (2001) Neovascularization of ischemic myocardium by human bone-marrow-derived angioblasts prevents cardiomyocyte apoptosis, reduces remodeling and improves cardiac function. Nat Med 7: 430–436. 11283669 10.1038/86498

[12] Davani S, Marandin A, Mersin N, Royer B, Kantelip B, et al (2003) Mesenchymal progenitor cells differentiate into an endothelial phenotype, enhance vascular density, and improve heart function in a rat cellular cardiomyoplasty model. Circulation 108S: 253–258. 10.1161/01.cir.0000089186.09692.fa 12970242

[13] Ziegelhoeffer T, Fernandez B, Kostin S, Heil M, Voswinckel R, et al (2004) Bone marrow-derived cells do not incorporate into the adult growing vasculature. Circ Res 94: 230–238. 14656934 10.1161/01.RES.0000110419.50982.1C

[14] O'Neill TT, Wamhoff BR, Owens GK, Skalak TC (2005) Mobilization of bone marrow-derived cells enhances the angiogenic response to hypoxia without transdifferentiation into endothelial cells. Circ Res 97: 1027–1035. 16210550 10.1161/01.RES.0000189259.69645.25

[15] Perry TE, Song M, Despres DJ, Kim SM, San H, et al (2009) Bone marrow-derived cells do not repair endothelium in a mouse model of chronic endothelial cell dysfunction. Cardiovasc Res 84: 317–325. 19578071 10.1093/cvr/cvp215 PMC2761200

[16] Zhang D, Huang W, Dai B, Zhao T, Ashraf A, et al (2010) Genetically manipulated progenitor cell sheet with diprotin A improves myocardial function and repair of infarcted hearts. Am J Physiol Heart Circ Physiol 299: H1339–H1347. 20802132 10.1152/ajpheart.00592.2010 PMC2993193

[17] Huang W, Zhang D, Millard RW, Wang T, Zhao T, et al (2010) Gene manipulated peritoneal cell patch repairs infarcted myocardium. J Mol Cell Cardiol 48: 702–712. 19913551 10.1016/j.yjmcc.2009.10.032 PMC2905838

[18] Zhang D, Fan GC, Zhou X, Zhao T, Pasha Z, et al (2008) Over-expression of CXCR4 on mesenchymal stem cells augments myoangiogenesis in the infarcted myocardium. J Mol Cell Cardiol 44: 281–292. 18201717 10.1016/j.yjmcc.2007.11.010 PMC2601571

[19] Ben-Shoshan J, Schwartz S, Luboshits G, Maysel-Auslender S, Barzelay A, et al (2008) Constitutive expression of HIF-1alpha and HIF-2alpha in bone marrow stromal cells differentially promotes their proangiogenic properties. Stem Cells 26: 2634–2643. 18687993 10.1634/stemcells.2008-0369

[20] Eun LY, Song BW, Cha MJ, Song H, Kim IK, et al (2010) Overexpression of phosphoinositide-3-kinase class II alpha enhances mesenchymal stem cell survival in infarcted myocardium. Biochem Biophys Res Commun 402: 272–279. 20937252 10.1016/j.bbrc.2010.10.013

[21] Xu J, Liu X, Jiang Y, Chu L, Hao H, et al (2008) MAPK/ERK signalling mediates VEGF-induced bone marrow stem cell differentiation into endothelial cell. J Cell Mol Med 12: 2395–2406. 18266967 10.1111/j.1582-4934.2008.00266.x PMC4514117

[22] Shabbir A, Zisa D, Lin H, Mastri M, Roloff G, et al (2010) Activation of host tissue trophic factors through JAK-STAT3 signaling: a mechanism of mesenchymal stem cell-mediated cardiac repair. Am J Physiol Heart Circ Physiol 299: H1428–H1438. 20852053 10.1152/ajpheart.00488.2010 PMC2993206

[23] Mullen CA (1994) Metabolic suicide genes in gene therapy. Pharmacol Ther 63: 199–207. 7809180 10.1016/0163-7258(94)90046-9

[24] Mavria G, Porter CD (2001) Reduced growth in response to ganciclovir treatment of subcutaneous xenografts expressing HSV-tk in the vascular compartment. Gene Ther 8: 913–920. 11426331 10.1038/sj.gt.3301483

[25] Cowan CE, Kohler EE, Dugan TA, Mirza MK, Malik AB, et al (2010) Kruppel-like factor-4 transcriptionally regulates VE-cadherin expression and endothelial barrier function. Circ Res 107: 959–966. 20724706 10.1161/CIRCRESAHA.110.219592 PMC3018700

[26] Dai B, Huang W, Xu M, Millard RW, Gao MH, et al (2011) Reduced collagen deposition in infarcted myocardium facilitates induced pluripotent stem cell engraftment and angiomyogenesis for improvement of left ventricular function. J Am Coll Cardiol 58: 2118–2127. 22051336 10.1016/j.jacc.2011.06.062 PMC3342759

[27] Wang Y, Zhang D, Ashraf M, Zhao T, Huang W, et al (2010) Combining neuropeptide Y and mesenchymal stem cells reverses remodeling after myocardial infarction. Am J Physiol Heart Circ Physiol 298: H275–H286. 19897711 10.1152/ajpheart.00765.2009 PMC2806133

[28] Caplan AI, Correa D (2011) The MSC: An Injury Drugstore. Cell Stem Cell 9: 11–15. 21726829 10.1016/j.stem.2011.06.008 PMC3144500

[29] Lee S, Chen TT, Barber CL, Jordan MC, Murdock J, et al (2007) Autocrine VEGF signaling is required for vascular homeostasis. Cell 130: 691–703. 17719546 10.1016/j.cell.2007.06.054 PMC3010851

[30] Walshe TE, D'Amore PA (2008) The role of hypoxia in vascular injury and repair. Annu Rev Pathol 3: 615–643. 18039132 10.1146/annurev.pathmechdis.3.121806.151501

[31] Mohyeldin A, Garzon-Muvdi T, Quinones-Hinojosa A (2010) Oxygen in stem cell biology: a critical component of the stem cell niche. Cell Stem Cell 7: 150–161. 20682444 10.1016/j.stem.2010.07.007

[32] Majmundar AJ, Wong WJ, Simon MC (2010) Hypoxia-Inducible Factors and the Response to Hypoxic Stress. Mol Cell 40: 294–309. 20965423 10.1016/j.molcel.2010.09.022 PMC3143508

[33] Nakayama K, Frew IJ, Hagensen M, Skals M, Habelhah H, et al (2004) Siah2 regulates stability of prolyl-hydroxylases, controls HIF1alpha abundance, and modulates physiological responses to hypoxia. Cell 117: 941–952. 15210114 10.1016/j.cell.2004.06.001

[34] Huber P, Dalmon J, Engiles J, Breviario F, Gory S, et al (1996) Genomic structure and chromosomal mapping of the mouse VE-cadherin gene (Cdh5). Genomics 32: 21–28. 8786117 10.1006/geno.1996.0072

[35] Gory S, Vernet M, Laurent M, Dejana E, Dalmon J, et al (1999) The vascular endothelial-cadherin promoter directs endothelial-specific expression in transgenic mice. Blood 93: 184–192. 9864160

[36] Ganju RK, Brubaker SA, Meyer J, Dutt P, Yang Y, et al (1998) The alpha-chemokine, stromal cell-derived factor-1alpha, binds to the transmembrane G-protein-coupled CXCR-4 receptor and activates multiple signal transduction pathways. J Biol Chem 273: 23169–23175. 9722546 10.1074/jbc.273.36.23169

[37] Zhang Y, Foudi A, Geay JF, Berthebaud M, Buet D, et al (2004) Intracellular localization and constitutive endocytosis of CXCR4 in human CD34+ hematopoietic progenitor cells. Stem Cells 22: 1015–1029. 15536192 10.1634/stemcells.22-6-1015

[38] Venkatesan S, Rose JJ, Lodge R, Murphy PM, Foley JF (2003) Distinct mechanisms of agonist-induced endocytosis for human chemokine receptors CCR5 and CXCR4. Mol Biol Cell 14: 3305–3324. 12925765 10.1091/mbc.E02-11-0714 PMC181569

[39] Huang C, Gu H, Zhang W, Manukyan MC, Shou W, et al (2011) SDF-1/CXCR4 mediates acute protection of cardiac function through myocardial STAT3 signaling following global ischemia/reperfusion injury. Am J Physiol Heart Circ Physiol 301: H1496–H1505. 21821779 10.1152/ajpheart.00365.2011 PMC3197365

[40] Iwakura T, Mohri T, Hamatani T, Obana M, Yamashita T, et al (2011) STAT3/Pim-1 signaling pathway plays a crucial role in endothelial differentiation of cardiac resident Sca-1+ cells both in vitro and in vivo. J Mol Cell Cardiol 51: 207–214. 21600215 10.1016/j.yjmcc.2011.04.013

[41] Le Bras A, Lionneton F, Mattot V, Lelievre E, Caetano B, et al (2007) HIF-2alpha specifically activates the VE-cadherin promoter independently of hypoxia and in synergy with Ets-1 through two essential ETS-binding sites. Oncogene 26: 7480–7489. 17563748 10.1038/sj.onc.1210566

[42] Gory S, Dalmon J, Prandini MH, Kortulewski T, de Launoit Y, et al (1998) Requirement of a GT box (Sp1 site) and two Ets binding sites for vascular endothelial cadherin gene transcription. J Biol Chem 273: 6750–6755. 9506975 10.1074/jbc.273.12.6750

[43] Yin L, Ohanyan V, Pung YF, Delucia A, Bailey E, et al (2012) Induction of vascular progenitor cells from endothelial cells stimulates coronary collateral growth. Circ Res 110: 241–252. 22095729 10.1161/CIRCRESAHA.111.250126 PMC3974272

[44] Herbert SP, Stainier DY (2011) Molecular control of endothelial cell behaviour during blood vessel morphogenesis. Nat Rev Mol Cell Biol 12: 551–564. 21860391 10.1038/nrm3176 PMC3319719

