# Supplementary material for: Suicide Gene Reveals the Myocardial Neovascularization Role of Mesenchymal Stem Cells Overexpressing CXCR4 (MSCCXCR4)
Source: PLoS One. 2012 Sep 28;7(9):e46158. doi: 10.1371/journal.pone.0046158 (PMC3460871; doi:10.1371/journal.pone.0046158)
Supplement: Table S1 — The sequences of PCR primers. F, forward primer; R, reverse primer. (DOCX) [file pone.0046158.s001.docx]

| Target | primer sequences |
| --- | --- |
| RT-PCR |  |
| CXCR4 | F: 5'-ACTCCCCTTCTGGGCAGTGGAC-3' |
|  | R: 5'-GTCGGGGTAAAGGCGGTCACAG-3' |
| VEGF-A | F: 5'-GGCAGCTTGAGTTAAACGAAC-3' |
|  | R: 5'-TGGTGACATGGTTAATCGGTC-3' |
| TK | F: 5'-CTCACCCTCATCTTCGACCG-3' |
|  | R: 5'-CCTGCAGATACCGCACCGTA-3' |
| IGF-1 | F: 5'-GAGACTGGAGATGTACTGTGC-3' |
|  | R: 5'-CTCCTTTGCAGCTTCGTTTTC-3' |
| VE-cadherin | F: 5'-TGGTCACCTACGACGAGGAGG-3' |
|  | R: 5'-GCCCTCGTAGCCGTAGATGTG-3' |
| Angiopoietin-1 | F: 5'-CAGATACAACAGAATGCGGTTCA-3' |
|  | R: 5'-TGAGACAAGAGGCTGGTTCCTAT-3' |
| CD31 | F: 5'-GCTGTCTACTCAGTCATGGCC-3' |
|  | R: 5'-CGTCTCTTCCTTCTGGATGGTG-3' |
| TK expression vector |  |
| VE-cadherin promoter | F: 5'-GGATCGATGCTTGCCCAGCTATATAATAAAACAAG-3' |
|  | R: 5'-AATCTAGAGAGCCTCTCTGTCATGGGAGT-3' |
| TK CDS | F: 5'-GCAGGAATTCATGGCCTCGTACCCCGGCCATCAA-3' |
|  | R: 5'-TTAATAGCGGCCGCTCAGTTAGCCTCCCCCATCTCC-3' |
| CXCR4 expression vector | |
| CXCR4 CDS | F: 5'-CGGCCCGAATTCATGGAAATATACACTTCGGATAAC-3' |
|  | R: 5'-ATTTGCGGCCGCTTAGCTGGAGTGAAAACTTGAG-3' |
